# Zika Virus Infection Disrupts Astrocytic Proteins Involved in Synapse Control and Axon Guidance

**DOI:** 10.3389/fmicb.2019.00596

**Published:** 2019-03-26

**Authors:** Affan A. Sher, Kathleen K. M. Glover, Kevin M. Coombs

**Affiliations:** ^1^Department of Medical Microbiology and Infectious Diseases, University of Manitoba, Winnipeg, MB, Canada; ^2^Manitoba Centre for Proteomics and Systems Biology, Winnipeg, MB, Canada; ^3^Children’s Hospital Research Institute of Manitoba, Winnipeg, MB, Canada

**Keywords:** RNA virus infection, proteomics, aptamers, SOMAScan, astrocytic dysregulation, virus-host interaction

## Abstract

The first human Zika virus (ZIKV) outbreak was reported in Micronesia in 2007, followed by one in Brazil in 2015. Recent studies have reported cases in Europe, Oceania and Latin America. In 2016, ZIKV transmission was also reported in the US and the World Health Organization declared it a Public Health Emergency of International Concern. Because various neurological conditions are associated with ZIKV, such as microcephaly, Guillain-Barré syndrome, and other disorders of both the central and peripheral nervous systems, including encephalopathy, (meningo)encephalitis and myelitis, and because of the lack of reliable patient diagnosis, numerous ongoing studies seek to understand molecular mechanisms underlying ZIKV pathogenesis. Astrocytes are one of the most abundant cells in the CNS. They control axonal guidance, synaptic signaling, neurotransmitter trafficking and maintenance of neurons, and are targeted by ZIKV. In this study, we used a newly developed multiplexed aptamer-based technique (SOMAScan) to examine > 1300 human astrocyte cell proteins. We identified almost 300 astrocyte proteins significantly dysregulated by ZIKV infection that span diverse functions and signaling pathways, including protein translation, synaptic control, cell migration and differentiation.

## Introduction

More than 3 billion people are at risk for infection by arthropod-borne viruses (arboviruses). Zika virus (ZIKV) is a newly re-emerging mosquito-borne virus that is known to cause rare but serious birth defects (De Carvalho et al., 2016; Teixeira et al., 2016). ZIKV is a flavivirus that contains a positive single-stranded RNA genome that encodes three structural (capsid, pre-membrane and envelope) and seven non-structural proteins (Peng et al., 2018). ZIKV was first isolated in 1947 from sentinel rhesus monkeys in the Zika forest near Entebbe, Uganda and has since spread to the Pacific and the Americas (Ioos et al., 2014; Barzon et al., 2016). ZIKV is transmitted by the mosquito vectors *Aedes aegypti* and *A. albopictus*. *A. aegypti* feeds on the blood of many hosts and in the process is able to spread ZIKV to numerous individuals, making it the main mosquito species involved in ZIKV transmission (Glover and Coombs, 2017). Sexual transmission also has been reported (Moreira et al., 2017).

The serious brain malformations in newborn babies infected with ZIKV *in utero* include microcephaly, cortical thinning and blindness, and reflect the ability of ZIKV to readily infect the developing human brain. ZIKV can successfully target and replicate in cells in different brain regions, including brain stem/progenitor cells of the proliferating ventricular zone (Gabriel et al., 2017), oligodendroglia and microglia, whereas neurons are less susceptible (Retallack et al., 2016). Recently, it was shown that ZIKV efficiently infects numerous cell types ranging from astrocytes, neural progenitor cells and microglia-like cells to Sertoli cells and human umbilical vein endothelial cells (Kozak et al., 2017; Siemann et al., 2017; Muffat et al., 2018; Peng et al., 2018). Astrocytes are a major brain cell population that are considered an essential first site of ZIKV infection (van den Pol et al., 2017). Astrocytes act as initiators and drivers of ZIKV infection within the developing brain. Thus, given the ability of this virus to infect not only brain cells and brain cell precursors, and its ability to penetrate the placental barrier, ZIKV infection results in various neurological conditions associated with the central nervous system (CNS) [(meningo)encephalitis, myelitis and acute disseminated encephalomyetis (ADEM)] and with the peripheral nervous system (PNS) (Guillain-Barré syndrome, chronic inflammatory demyelinating polyneuropathy and acute transient polyneuritis) (Zhang et al., 2017; Leonhard et al., 2018; Mehta et al., 2018). Many of these clinical abnormalities are associated with problems occurring during the development of the brain in the fetus or in adult CNS and PNS. Of these two, CNS abnormalities have been associated with problems involving astrocytes (Wishart et al., 2006). Since astrocytes are highly involved in the survival of neurons, in axonal and synaptic guidance, and in coordination with other microglia in postnatal synaptogenesis, astrocytic death or functional disruption can result in numerous neurological conditions (Jacobsen and Miller, 2003; Gonzalez-Perez and Quinones-Hinojosa, 2012; Reemst et al., 2016). Astrocytes also migrate and assist in the migration of new neurons via the organization of the rostral migratory stream during early developmental stages (Bozoyan et al., 2012).

Since viruses are obligate intracellular parasites, they depend upon host cells and processes for replication. Numerous studies have demonstrated profound virus-induced alterations in the transcriptomic and proteomic profiles of virus-infected cells and organisms (for examples: Kobasa et al., 2007; Vester et al., 2009). Similar recent studies have identified ZIKV-induced changes in expression of large numbers of cellular genes and proteins. For example, Garcez et al. (2016) examined proteomic and transcriptomic responses to ZIKV infection in neurospheres and Kumar et al. (2018) measured transcriptomic alterations induced in Sertoli cells. Other global proteomic non-biased mass spectrometry-based approaches have been performed on ZIKV-infected C6/36 mosquito cells (Xin et al., 2017) and on neural progenitor cells (Jiang et al., 2018). Scaturro et al. (2018) recently performed an integrated affinity and phosphoproteomic analysis of human neural progenitor cells. Despite mass spectrometry being a powerful tool to identify and measure large numbers of proteins (reviewed in Yates et al., 2009; Coombs, 2011), drawbacks include that low abundant proteins may not be detected and there often is poor overlap between experimental replicates. We recently used a complementary newly developed multiplexed aptamer-based technique to measure > 1300 proteins in monkey Vero cells that had been infected with ZIKV for different amounts of time to determine temporal patterns of ZIKV-induced host protein dysregulation (Glover et al., 2019). Here, we used these same slow off-rate modified aptamers (SOMAmers^®^; SomaLogics, Inc., Denver, CO, United States; SOMAScan, 2017) to assay alterations in the levels of ZIKV-induced protein expression in human glioblastoma U251 cells as a model for these more physiologically relevant human astrocyte cells. We identified 170 unique U-251 proteins that were significantly over- or under-expressed at least 1.5-fold compared to non-infected that belong to several broad cellular networks, including apoptosis and pathways important for astrocytic cell synaptic control, cell growth and migration.

## Materials and Methods

### Cells and Viruses

#### Cells

Human U-251 glioblastoma astrocytoma [U-251 MG (formerly known as U-373 MG; European Collection of Authenticated Cell Cultures – ECACC 09063001)] were cultured in Dulbecco’s modified Eagle’s/Nutrient Mixture F-12 (DMEM/F-12) medium. Monkey Vero cells (ATCC^®^ # CCL-81^TM^) were cultured in Dulbecco’s modified Eagle’s medium (DMEM). Both media were supplemented with 10% Fetal Bovine Serum (FBS), non-essential amino acids, 2 mM L-glutamine, and sodium pyruvate, respectively, and cells were maintained at 37°C in 5% CO_2_. Cells were trypsinized and passaged every 2 – 3 days.

#### Virus

The Asian strain of ZIKV was a gift from Dr. David Safronetz, Chief of Special Pathogens, the National Microbiology Laboratory, Public Health Agency of Canada. Virus was propagated in Vero cells in DMEM supplemented with 2.5% FBS, non-essential amino acids, 2 mM L-glutamine, sodium pyruvate, 2× gentamicin and 2× Amphotericin B at 37°C in 5% CO_2_.

### ZIKV Infections

#### Stock Virus Growth

Vero cells were grown to 60–70% confluency in fully supplemented DMEM media and infected with ZIKV at MOI ∼ 0.001 PFU/cell. Media were replaced after virus adsorption with DMEM supplemented as above but containing 2.5% FBS. Supernatants were collected at different time-points to harvest virus, centrifuged at 600 ×*g* for 8 min at 4°C to remove cell debris, supplemented to 20% FBS and frozen at -80°C until used later for titration and U251 infections.

#### ZIKV Titration

Vero cells were grown to 60–70% confluency in 6-well plates. Serial 10-fold dilutions of viral stocks were made in gel saline [137 mM NaCl, 0.2 mM CaCl_2_, 0.8 mM MgCl_2_, 19 mM HBO_3_, 0.1 mM Na_2_B_4_O_7_, 0.3% (wt/vol) gelatin], and adsorbed in duplicate on the Vero cell monolayers. After viral attachment (2 h in a 37°C CO_2_ incubator with periodic rocking), 2.5 ml per well of a 50:50 (vol/vol) mixture of [1.2% Type I Agarose (Difco Laboratories, Detroit, Mich.)] and [2 × completed Medium 199 (Medium 199 with 6% FBS, 4 mM glutamine, 10 μg of gentamicin sulfate per ml, and 3 μg of amphotericin B per ml)] was added to each well to overlay cells. Cells were incubated at 37°C for 5 days and then overlaid with 1% Bacto Agar in 1× PBS that contained 0.04% neutral red. Plaques were counted 18–21 h after neutral red staining.

#### Experimental Infections

U-251 cells were grown to 60-70% confluency in fully supplemented DMEM/F-12 media and infected at MOI = 3, which, by Poisson calculation, suggests > 95% of cells are infected. Virus was adsorbed in gel saline as described above for 2 h in a 37°C CO_2_ incubator with periodic rocking. Mock-infected cells were treated similarly in gel saline that contained no virus. After adsorption, cells were overlaid with DMEM/F12 supplemented as described above but containing 2.5% FBS and incubated at 37°C for various periods of time as indicated below.

### Photomicroscopy

#### Bright-Field Microscopy

Infected- and mock-treated U-251 cells were microscopically observed for cytopathic effect (CPE) at 6, 12, 24, 48, and 72 h post-infection (hpi) using a Nikon TE-2000 inverted microscope. All images were taken with a Canon A-700 digital camera and exported into PowerPoint for minimal adjustment of brightness and contrast.

#### Immunofluorescence Microscopy

U-251 cells were seeded on spotted slides at 20,000 cells/spot, infected at an MOI = 3 as described above and processed at 12, 24, and 48 hpi. Cells were fixed using 4% paraformaldehyde for 15 min, followed by permeabilization with 0.1% Triton X-100 in PBS for 5 min. Cells were then blocked in 3% BSA blocking solution for 90 min, followed by overnight incubation at 4°C with mouse anti-ZIKV NS1 primary antibody (BioFront Technologies; cat # BF-1225-06) in 1% BSA and PBS. Cells were washed with PBT buffer (PBS with 0.2% Tween-20) and secondary anti-mouse antibody tagged with Alexa Fluor^TM^ 488 (Invitrogen #A-11001) was added for 1 h. Slides were washed 3× with PBT buffer followed by mounting using DAPI-Prolong^®^ Gold AntiFade to stain nuclei. Slide edges were sealed using nail polish and the slides kept at 4°C. until visualized. Images were taken with a Zeiss Axio Observer ZI inverted microscope using 20× objective. AxioVision 4.8.2 software was used to capture and edit the images before exporting them.

### Cell Viability

Cell viability was measured using the WST-1 assay according to manufacturer’s instructions (Roche), but adding 8 μL of WST-1 reagent instead of 10 μL into each well of a 96-well plate at desired times. The cells were incubated for 2 h at 37°C and then absorbance was measured at 440 and 610 nm. Absorbance at 610 nm was subtracted from that at 440 nm and the values of the ZIKV-infected samples were normalized with their respective mock samples at each time point. A minimum of triplicate experiments were analyzed.

### Protein Quantification

Mock- and ZIKV-infected U-251 samples were harvested at each of various time points (12, 24, and 48 hpi) and washed 3× with >50-volumes of PBS to remove media and culture FBS. Washed cells were lysed with MPER^®^ (Pierce; Rockford, IL, United States) supplemented with 1× HALT^®^ Protease inhibitor (Pierce; Rockford, IL, United States). After cell lysis, lysates were centrifuged at 14,000 ×*g* for 15 min at 11°C to remove insoluble cellular components. Cell lysate protein quantities were determined by BCA^TM^ Protein Assay (Pierce; Rockford, IL, United States) and normalized to bovine serum albumin standards.

### SOMAScan^®^ Analyses

Protein concentrations of BCA-determined cell lysates were adjusted to 200 ng/μL, and 70 μL of each sample submitted for SOMAScan^®^ analysis in-house on a SomaLogics^®^-licensed platform in the Manitoba Centre for Proteomics and Systems Biology as described (SOMAScan, 2017 #14453; Glover et al., 2019). Briefly, the SOMAScan assay is a novel proteomic tool that uses single-stranded DNA-based Slow Off-rate Modified Aptamer reagents (SOMAmers). These chemically modified nucleotides were selected based upon their capacity to bind to specific human proteins. The SOMAmers capture proteins in their native state, and, after a series of washing steps, are released and their quantities measured on DNA microarray chips. When the SOMAmers are used to probe a range of sample concentrations, they are capable of measuring femtomolar to micromolar quantities of proteins. We used the SOMAScan version 1.3, capable of simultaneously measuring 1,307 distinct proteins in each of up to 92 samples (SOMAScan, 2017). Three biologic replicates of infected samples collected at 12, 24, and 48 hpi, and of each time-matched mock-infected control (=18 total samples) were simultaneously analyzed in a single SOMAScan 96-well plate. Results were reported in Relative fluorescent units (RFU) for each sample, which are directly proportional to the amounts of target protein quantities in the initial samples, as confirmed by a standard curve generated for each protein-SOMAmer pair (SOMAScan, 2017). RFU differences between Mock and ZIKV-infected were analyzed as described below in Statistical and bioinformatics analyses.

### Immunoblotting

BCA-quantified protein samples were adjusted to load 10 μg of protein per gel lane. Samples were heated to 95°C for 5 min and resolved by mini-10% sodium dodecyl sulfate polyacrylamide electrophoresis (SDS-PAGE) until the loading dye had just run off the bottom edge of the gel. The proteins were transferred to PVDF [Immobilon-P polyvinylidene difluoride membrane (Millipore)] for 2 h in ice-cold buffer, followed by overnight blocking of the membrane in 5% skim milk in 1× TBST. Primary antibodies were added to each blot at 1:1000 dilution in 1% milk/TBST overnight. Primary antibodies used were: mouse monoclonal anti-ZIKV NS1 (BioFront Technologies # BF-1225-06); anti-ZIKV NS3 (GeneTex Inc. # GTX133309), anti-ZIKV envelope protein (GeneTex Inc. # GTX133314), anti-STAT3 (Cell Signaling # 124H6), anti-β-actin (Cell Signaling # 8H10D10); rabbit polyclonal anti-fibronectin (Abcam # ab2413), anti-SPARC (Cell Signaling # 5420S), and anti-cystatin C (Abcam # ab109508). After overnight binding, membranes were washed 3× with TBST and appropriate goat HRP-conjugated anti-rabbit (Cell Signaling # 7074) or anti-mouse (Cell Signaling # 7076) secondary antibodies were added for 1 h. The blots were washed 3 additional times with 1× TBST, developed with ECL western blotting peroxidase substrate for chemiluminescence and imaged with an enhanced chemiluminescence (ECL) detection machine (Amersham-Pharmacia Biotech); ImageJ was used to analyze each blot and each band in each blot was normalized to its respective actin control and to its time-matched mock-infected band intensity.

### Statistical and Bioinformatics Analyses

Relative fluorescent units values for each of the 1322 analytes (1307 unique proteins + 15 internal SOMA control analytes) in each of 3 biologic replicates, each consisting of a ZIKV-infected sample and a time-matched non-infected mock sample at 12, 24, and 48 hpi were imported into Excel and converted to Log_2_ values. Fold-changes were determined for each of the nine infected samples compared to their time-matched mock samples. The fold-changes were analyzed for significance by both Students *T*-test with 2 tails, and by *Z*-score analysis, as described (Coombs et al., 2010; Glover et al., 2019). Briefly, all fold-changes not deemed to be significant by *T*-test were examined by *Z*-score, expressing each value as its number of standard deviations away from the population mean. Each protein’s *Z*-score was considered significant if the average *Z*-score for that protein was > 1.96σ or <-1.96σ; and if the *Z*-score was < -1.96σ in each of 2 or more replicates and was < -0.98σ in no more than a single replicate, or if the *Z*-score was > 1.96σ in each of ≥ 2 replicates and was > 0.98σ in the remaining replicate. For increased stringency, we also applied a fold-change cut-off of 1.50-fold dysregulation (= ≥ 1.50-fold if up-regulated, or ≤0.667-fold if down-regulated) to those proteins considered significantly dysregulated. Fold changes and *P*-values were imported into DAVID, Network Analyst and Ingenuity Pathway Analysis (IPA^®^) for additional bioinformatics and pathway analyses. Western blot data were examined for significance by one-way ANOVA, using a significance cut-off of 0.05.

## Results

### ZIKV Successfully Establishes Infection in U-251 Cells

**FIGURE 1:**
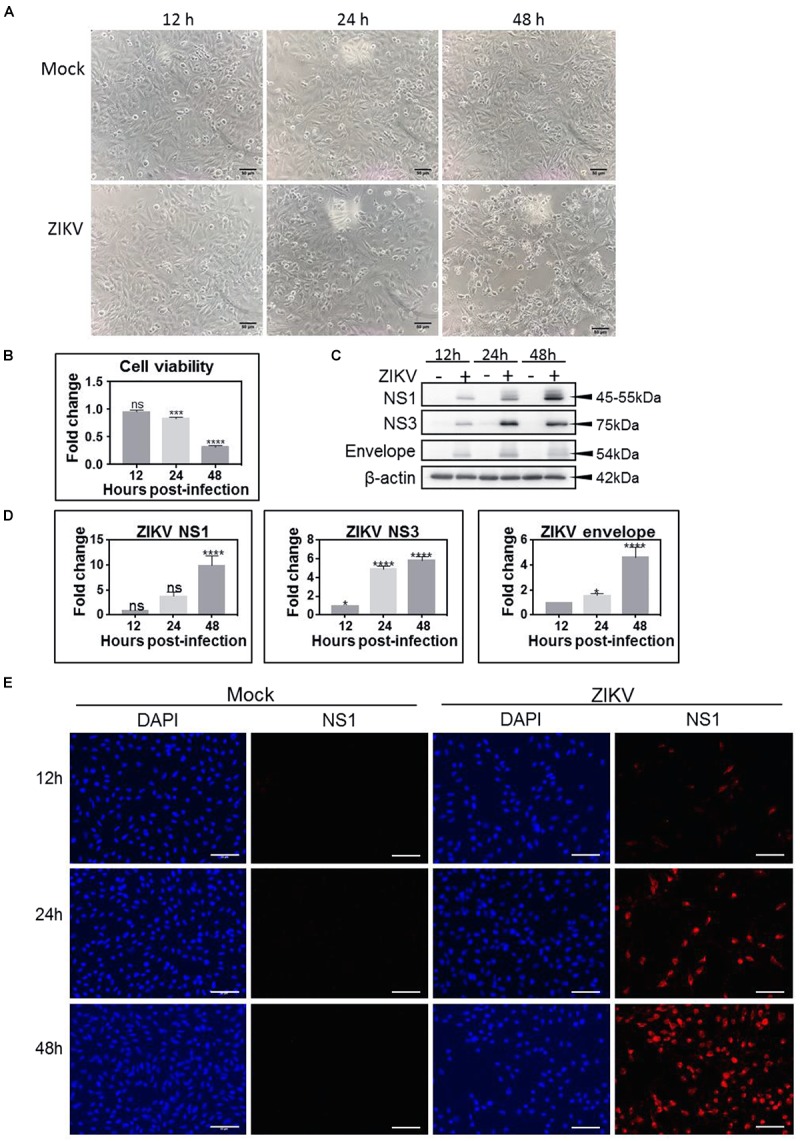
Zika virus (ZIKV) productively infects U-251 astrocyte cells. **(A)** Bright-field micrograph at 100× magnification shows induction of cytopathic effect (CPE) in ZIKV-infected U-251 cells (bottom) over time compared to mock (top). **(B)** Cell viability of ZIKV-infected U-251 cells, as measured by the WST-1 assay over time and compared to mock-treated cells. **(C)** Western blot analyses of ZIKV non-structural (NS)1, NS3 and envelope protein production in U-251 cells over time. **(D)** Densitometric analyses of Western blots such as depicted in **(C)**. **(E)** Immunofluorescent detection of proportions of ZIKV-infected cells. U-251 cells were mock-treated, or infected at an MOI = 3 for indicated times and immunoprobed to determine proportions of productively infected cells. DAPI (blue) for nuclei and Alexa 546 (red) for ZIKV NS1. Scale bars are 50 μm. Significance levels: ^∗^*p* < 0.05; ^∗∗∗^*p* < 0.002; ^∗∗∗∗^*p* < 0.001.

Unlike the mock-treated astrocytic U-251 cells, ZIKV-infected astrocytes show a visible cytopathic effect (CPE) over time as they start to circularize and lift from the surface (Figure 1A), and increasing CPE with time post-infection was measured by cell viability assays (Figure 1B). ZIKV also promotes increasing production of its viral non-structural proteins, NS1 and NS3, along with structural protein, envelope, as time progresses (Figure 1C). Densitometric analyses of each of these viral proteins shows the first signal by 12 hpi (Figure 1D). NS3 protein gives the strongest signal at 24 hpi (Figure 1D). By 48 hpi, all tested viral proteins show 4 – 10-fold increase in expression (Figure 1D). We also used immunofluorescence microscopy to probe expression of viral non-structural protein NS1, which is only expressed during successful infection, to confirm that >90% of cells were infected by 48 hpi (Figure 1E), which allows subsequent meaningful proteomic analyses.

### ZIKV Alters the Proteomic State of U-251 Cells With Increasing Amounts of Dysregulation as the Time After Infection Increases

**FIGURE 2:**
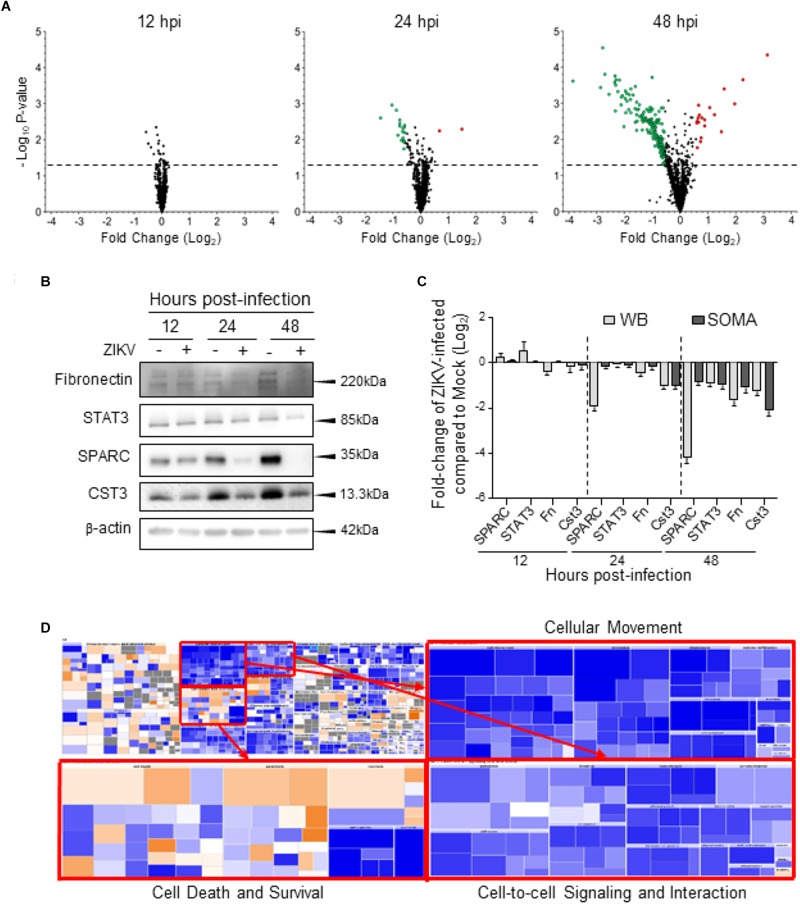
Global dysregulation of U-251 proteins and cellular processes. **(A)** Volcano plots displaying *p*-values and Log_2_-fold changes of each protein at each time point after compiling and merging data from all three biologic replicates. Larger red dots correspond to proteins considered significantly up-regulated (fold-change ≥ 1.5; Log_2_ ≥ 0.585) and larger green dots correspond to proteins considered significantly down-regulated (fold-change ≤ 0.667 compared to mock; Log_2_ ≤ –0.585). The horizontal dashed lines correspond to *p*-value of 0.05. **(B)** Western blot validation of indicated proteins. Cellular proteins were harvested from ZIKV-infected U-251 cells (+), or from mock-infected (–) cells at indicated times after MOI = 3 infection, resolved by SDS-PAGE, transferred to nitrocellulose membranes, and membranes probed with indicated antibodies. These blots are representative of three biologic replicates. Molecular weight marker locations shown at right. **(C)** Densitometric comparisons of Western blot data such as that shown in “B” to SOMAScan-derived protein dysregulation. **(D)** Impact of ZIKV at 48 h post infection (hpi) on the whole cellular Biofunctions Map (upper left), determined by IPA. The three major categories; Cellular Movement, Cell death and survival, and Cell-to-cell signaling and interaction are expanded to highlight numbers of dysregulated molecules (each colored block).

We analyzed the cellular proteome of ZIKV-infected U-251 astrocytes at 12, 24, and 48 hpi and compared them to time-matched mock-treated cells using the novel SOMAScan platform. A total of 296 cellular proteins were significantly over- or under-expressed compared to mock, non-infected, with 17 over-expressed and 17 under-expressed at 12 hpi, 23 over- and 40 under-expressed at 24 hpi, and 45 over- and 209 under-expressed at 48 hpi (Table 1). For further analysis, we also consider each protein’s fold-change along with its significance. We set the cut-offs as *P*-value < 0.05 and fold-change of ≥ 1.50 [over-expressed to ≥ 1.50 (≥0.585 Log_2_) or under-expressed to ≤0.667 (≤-0.585 Log_2_)]. This cut-off value was chosen to keep the numbers of significant differentially expressed molecules large enough to perform meaningful bioinformatics analyses, while applying sufficient stringency to maintain physiologic relevance. Using these criteria, no proteins were significantly over- or under-expressed at 12 hpi, 2 proteins were significantly up-regulated and 17 were significantly down-regulated at 24 hpi, and 18 proteins were significantly up-regulated and 151 were significantly down-regulated at 48 hpi (Table 1 and Figure 2A). The 170 unique proteins significantly over- or under-expressed ≥ 1.50-fold at any of the tested time points (12, 24, or 48 hpi) are listed and bolded in Table 2.

**Table 1:** Numbers of significantly dysregulated ZIKV-infected U-251 proteins.

Number that are significant	Total Unique	12 hpi	24 hpi	48 hpi
and fold-change > 1.000	296	17	23	45
and fold-change < 0.9999		17	40	209
and fold-change > 1.100	272	2	21	43
and fold-change < 0.9091		8	40	209
and fold-change > 1.250	244	0	10	30
and fold-change < 0.8000		3	37	204
and fold-change > 1.500	**170**	**0**	**2**	**18**
and fold-change < 0.6667		**0**	**17**	**151**
and fold-change > 1.750	113	0	1	9
and fold-change < 0.5714		0	4	102
and fold-change > 2.000	80	0	1	7
and fold-change < 0.5000		0	2	73


*Significance was determined by *T*-test and *Z*-score as detailed in Section “Materials and Methods.” Values that are bolded correspond to significant differential expression and fold-change ≥ 1.50 that are listed in Table 2 and that were used for subsequent bioinformatics analyses.*

**Table 2:** U-251 proteins significantly dysregulated by ZIKV infection.

		12 hpi		24 hpi		48 hpi	
Entrez Gene Symbol	Protein	Fold Change	*P*-value	Fold Change	*P*-value	Fold Change	*P*-value
*Significantly up-regulated proteins*							
CXCL11	C-X-C motif chemokine 11	0.865	**0.005**	**2.763**	**0.005**	**8.614**	**4.4E-05**
NACA	Nascent polypeptide-associated complex subunit alpha	1.040	0.281	**1.581**	**0.006**	0.974	0.761
CCL5	C-C motif chemokine 5	1.041	0.178	1.316	**0.032**	**4.721**	**2.1E-04**
CKB	Creatine kinase B-type	0.989	0.860	0.988	0.931	**3.847**	**0.001**
SSRP1	FACT complex subunit SSRP1	0.995	0.968	1.422	**0.040**	**2.951**	**3.9E-04**
ISG15	Ubiquitin-like protein ISG15	0.998	0.982	1.261	**0.039**	**2.749**	**0.006**
HMGN1	Non-histone chromosomal protein HMG-14	1.016	0.815	1.169	0.143	**2.312**	**0.002**
NUDCD3	NudC domain-containing protein 3	0.986	0.738	1.135	0.125	**2.055**	**0.001**
PLCG1	1-phosphatidylinositol 4,5-bisphosphate phosphodiesterase gamma-1	1.015	0.775	1.183	**0.043**	**1.818**	**0.004**
METAP2	Methionine aminopeptidase 2	0.992	0.947	1.118	0.076	**1.812**	**0.003**
ADSL	Adenylosuccinate lyase	1.094	0.101	1.192	0.143	**1.694**	**0.002**
PARK7	Protein deglycase DJ-1	1.028	0.326	1.225	0.057	**1.665**	**0.009**
HSPA1A	Heat shock 70 kDa protein 1A	1.160	**0.042**	1.224	**0.019**	**1.642**	**0.011**
GDI2	Rab GDP dissociation inhibitor beta	1.065	0.512	1.114	0.111	**1.593**	**0.002**
PEBP1	Phosphatidylethanolamine-binding protein 1	0.993	0.942	1.169	0.189	**1.564**	**0.003**
UBE2N	Ubiquitin-conjugating enzyme E2 N	1.047	0.585	1.101	0.197	**1.557**	**0.001**
PPA1	Inorganic pyrophosphatase	1.035	0.684	1.139	0.075	**1.534**	**0.002**
IFNL1	Interferon lambda-1	0.908	0.060	1.239	**0.017**	**1.512**	**0.016**
EIF5A	Eukaryotic translation initiation factor 5A-1	1.040	0.653	1.143	**0.039**	**1.505**	**0.003**
*Significantly down-regulated proteins*							
STC1	Stanniocalcin-1	0.902	0.437	**0.363**	**0.002**	**0.069**	**2.3E-04**
CST3	Cystatin-C	0.913	0.445	**0.482**	**0.001**	**0.232**	**0.001**
IGFBP2	Insulin-like growth factor-binding protein 2	0.976	0.689	**0.537**	**0.001**	**0.213**	**4.6E-04**
SFRP1	Secreted frizzled-related protein 1	0.904	0.211	**0.562**	**0.007**	**0.137**	**3.5E-04**
APP	Amyloid beta A4 protein	0.976	0.786	**0.585**	**0.003**	**0.154**	**1.5E-04**
MFGE8	Lactadherin	0.946	0.410	**0.587**	**0.003**	**0.229**	**0.001**
MDK	Midkine	1.019	0.629	**0.588**	**0.004**	**0.199**	**0.002**
IGFBP7	Insulin-like growth factor-binding protein 7	0.919	0.256	**0.611**	**0.010**	**0.237**	**0.001**
IGFBP5	Insulin-like growth factor-binding protein 5	0.982	0.767	**0.630**	**0.006**	**0.145**	**2.8E-05**
CTSA	Lysosomal protective protein	0.946	0.353	**0.637**	**0.005**	**0.281**	**0.001**
LRIG3	Leucine-rich repeats and immunoglobulin-like domains protein 3	0.978	0.688	**0.638**	**0.009**	**0.203**	**2.2E-04**
FGFR1	Fibroblast growth factor receptor 1	0.937	0.291	**0.648**	**0.011**	**0.200**	**3.0E-04**
PCSK9	Proprotein convertase subtilisin/kexin type 9	1.012	0.840	**0.653**	**0.017**	**0.221**	**2.2E-04**
CFI	Complement factor I	0.911	0.342	**0.655**	**0.012**	**0.170**	**0.001**
TIMP2	Metalloproteinase inhibitor 2	0.999	0.986	**0.657**	**0.004**	**0.327**	**3.3E-04**
FSTL1	Follistatin-related protein 1	1.012	0.769	**0.658**	**0.010**	**0.261**	**0.001**
GRN	Granulins	0.961	0.578	**0.662**	**0.004**	**0.198**	**1.7E-04**
HS6ST1	Heparan-sulfate 6-*O*-sulfotransferase 1	0.964	0.241	0.710	**0.007**	**0.196**	**2.5E-04**
C4A C4B	Complement C4	0.916	0.265	1.044	0.711	**0.246**	**0.004**
PLAU	Urokinase-type plasminogen activator	0.972	0.530	0.779	**0.041**	**0.253**	**0.001**
TNC	Tenascin	0.973	0.480	0.732	**0.010**	**0.257**	**4.4E-04**
GPNMB	Transmembrane glycoprotein NMB	1.014	0.797	0.914	0.419	**0.258**	**0.002**
GFRA1	GDNF family receptor alpha-1	0.971	0.638	0.744	**0.016**	**0.267**	**0.001**
LAMA1 LAMB1 LAMC1	Laminin	0.949	0.319	0.762	**0.019**	**0.277**	**0.001**
THBS2	Thrombospondin-2	0.930	0.133	0.710	**0.016**	**0.290**	**0.001**
ACVR1B	Activin receptor type-1B	1.001	0.990	1.142	0.194	**0.293**	**0.002**
XRCC6	X-ray repair cross-complementing protein 6	0.936	0.222	0.906	0.357	**0.293**	**3.9E-04**
GNS	*N*-acetylglucosamine-6-sulfatase	1.004	0.948	0.878	0.179	**0.309**	**0.001**
CCL13	C-C motif chemokine 13	1.003	0.958	1.084	0.331	**0.326**	**0.001**
CCL20	C-C motif chemokine 20	0.969	0.686	1.100	0.421	**0.327**	**0.003**
L1CAM	Neural cell adhesion molecule L1	0.900	0.187	0.880	0.070	**0.336**	**0.001**
IGF1R	Insulin-like growth factor 1 receptor	1.018	0.624	0.972	0.669	**0.338**	**0.005**
ICAM5	Intercellular adhesion molecule 5	0.971	0.494	0.939	0.428	**0.338**	**0.002**
PLXNB2	Plexin-B2	0.974	0.604	0.826	**0.031**	**0.342**	**0.001**
GSN	Gelsolin	0.913	0.312	0.899	0.294	**0.344**	**0.003**
C4A C4B	Complement C4b	0.958	0.570	1.115	0.336	**0.352**	**0.001**
IL17RA	Interleukin-17 receptor A	0.951	0.541	0.930	0.445	**0.361**	**0.001**
LYN	Tyrosine-protein kinase Lyn, isoform B	0.956	0.635	0.904	0.450	**0.364**	**0.002**
PF4	Platelet factor 4	0.981	0.702	1.118	0.192	**0.374**	**0.002**
PROK1	Prokineticin-1	0.986	0.806	1.139	0.136	**0.385**	**0.003**
HIST1H1C	Histone H1.2	0.923	0.289	0.821	0.179	**0.390**	**0.005**
RTN4R	Reticulon-4 receptor	0.993	0.772	0.906	0.319	**0.392**	**0.002**
UNC5C	Netrin receptor UNC5C	1.004	0.875	0.837	0.086	**0.392**	**0.002**
LIFR	Leukemia inhibitory factor receptor	0.955	0.593	0.686	**0.012**	**0.396**	**0.003**
LYN	Tyrosine-protein kinase Lyn	0.925	0.387	0.897	0.429	**0.398**	**0.003**
FGF12	Fibroblast growth factor 12	0.981	0.709	1.061	0.512	**0.404**	**0.002**
HSD17B10	3-hydroxyacyl-CoA dehydrogenase type-2	0.984	0.688	0.991	0.917	**0.423**	**0.003**
CMA1	Chymase	0.989	0.804	1.126	0.141	**0.424**	**0.003**
CRISP3	Cysteine-rich secretory protein 3	0.994	0.934	1.122	0.208	**0.425**	**0.002**
ALB	Serum albumin	0.998	0.965	1.104	0.295	**0.434**	**0.001**
TNFRSF1A	Tumor necrosis factor receptor superfamily member 1A	1.005	0.904	0.783	**0.046**	**0.441**	**0.003**
SRC	Proto-oncogene tyrosine-protein kinase Src	0.977	0.706	0.907	0.353	**0.442**	**0.005**
FER	Tyrosine-protein kinase Fer	0.982	0.790	1.226	0.527	**0.452**	**0.006**
TFPI	Tissue factor pathway inhibitor	0.948	0.216	0.715	**0.013**	**0.458**	**0.002**
DYNLL1	Dynein light chain 1, cytoplasmic	0.990	0.823	1.095	0.308	**0.459**	**0.003**
MMP2	72 kDa type IV collagenase	0.983	0.729	0.877	0.103	**0.460**	**0.003**
RNASEH1	Ribonuclease H1	0.976	0.709	1.006	0.957	**0.463**	**0.003**
FN1	Fibronectin	1.013	0.750	0.851	0.075	**0.464**	**0.003**
SERPINA3	Alpha-1-antichymotrypsin	0.945	0.176	1.053	0.596	**0.467**	**0.003**
NRXN3	Neurexin-3-beta	0.983	0.479	0.918	0.281	**0.468**	**0.006**
PON1	Serum paraoxonase/arylesterase 1	0.971	0.598	1.098	0.347	**0.472**	**0.004**
FSTL3	Follistatin-related protein 3	0.910	0.370	0.727	**0.046**	**0.473**	**0.001**
NID1	Nidogen-1	0.977	0.641	0.843	0.066	**0.475**	**0.001**
B2M	Beta-2-microglobulin	0.969	0.580	0.721	**0.014**	**0.485**	**0.007**
UNC5D	Netrin receptor UNC5D	0.935	0.362	0.869	0.239	**0.486**	**0.002**
AGER	Advanced glycosylation end product-specific receptor, soluble	1.012	0.854	1.092	0.351	**0.489**	**0.0046**
MICB	MHC class I polypeptide-related sequence B	1.009	0.875	0.978	0.774	**0.489**	**0.005**
ECM1	Extracellular matrix protein 1	0.981	0.655	0.763	**0.019**	**0.489**	**0.003**
LY9	T-lymphocyte surface antigen Ly-9	1.009	0.925	1.139	0.080	**0.493**	**0.003**
ADAM12	Disintegrin and metalloproteinase domain-containing protein 12	0.988	0.764	0.952	0.616	**0.493**	**0.004**
PTN	Pleiotrophin	1.031	0.548	0.769	**0.026**	**0.496**	**0.004**
HIST1H3A	Histone H3.1	0.994	0.929	0.761	**0.010**	**0.498**	**1.8E-04**
TF	Serotransferrin	0.903	0.206	1.100	0.386	**0.499**	**0.001**
NOTCH1	Neurogenic locus notch homolog protein 1	0.987	0.854	0.877	0.215	**0.506**	**0.005**
CDON	Cell adhesion molecule-related/down-regulated by oncogenes	0.954	0.263	1.010	0.906	**0.507**	**0.008**
STAT3	Signal transducer and activator of transcription 3	1.018	0.770	0.903	0.178	**0.508**	**0.003**
CTSV	Cathepsin L2	0.990	0.777	0.714	**0.049**	**0.511**	**0.009**
C5	C5a anaphylatoxin	1.008	0.903	1.122	0.172	**0.511**	**0.007**
KIF23	Kinesin-like protein KIF23	0.930	0.182	0.884	0.066	**0.520**	**0.006**
BOC	Brother of CDO	0.931	0.314	1.077	0.619	**0.526**	**0.004**
IL15RA	Interleukin-15 receptor subunit alpha	0.992	0.862	1.137	0.169	**0.528**	**0.006**
GPC2	Glypican-2	1.005	0.895	1.148	0.303	**0.532**	**0.015**
SERPINE2	Glia-derived nexin	0.998	0.967	1.058	0.500	**0.532**	**0.006**
ANGPT2	Angiopoietin-2	0.971	0.501	1.137	0.136	**0.533**	**0.007**
F11	Coagulation Factor XI	1.006	0.899	1.110	0.235	**0.534**	**0.006**
CTSS	Cathepsin S	1.007	0.894	1.093	0.259	**0.535**	**0.006**
COL18A1	Endostatin	0.992	0.759	0.781	**0.024**	**0.536**	**0.003**
PRKACA	cAMP-dependent protein kinase catalytic subunit alpha	1.060	0.071	1.046	0.608	**0.536**	**0.002**
SPARC	SPARC	1.092	0.116	0.867	0.105	**0.537**	**0.004**
PLAT	Tissue-type plasminogen activator	0.992	0.780	0.885	0.145	**0.540**	**0.005**
PRKCA	Protein kinase C alpha type	0.946	0.296	1.018	0.925	**0.541**	**0.004**
THBS1	Thrombospondin-1	1.014	0.792	0.905	0.402	**0.552**	**0.010**
LGALS8	Galectin-8	0.979	0.640	0.813	**0.031**	**0.555**	**0.008**
AKT1 AKT2 AKT3	RAC-alpha/beta/gamma serine/threonine-protein kinase	0.960	0.526	0.973	0.780	**0.557**	**0.003**
KLK13	Kallikrein-13	1.011	0.833	1.111	0.200	**0.560**	**0.009**
POSTN	Periostin	0.988	0.852	1.161	0.230	**0.561**	**0.007**
MRC2	C-type mannose receptor 2	0.975	0.455	1.120	0.389	**0.566**	**0.014**
ICOS	Inducible T-cell costimulator	1.010	0.849	1.099	0.305	**0.569**	**0.008**
PDK1	[Pyruvate dehydrogenase (acetyl-transferring)] kinase isozyme 1, mitochondrial	1.025	0.587	1.075	0.244	**0.569**	**0.008**
DKK1	Dickkopf-related protein 1	0.916	**0.020**	0.748	**0.017**	**0.569**	**0.010**
METAP1	Methionine aminopeptidase 1	0.994	0.956	1.030	0.805	**0.570**	**0.003**
DDC	Aromatic-L-amino-acid decarboxylase	0.950	0.281	1.079	0.349	**0.570**	**0.003**
MAP2K3	Dual specificity mitogen-activated protein kinase kinase 3	1.011	0.609	1.087	0.269	**0.573**	**0.012**
CAMK2D	Calcium/calmodulin-dependent protein kinase type II subunit delta	0.997	0.963	1.086	0.577	**0.575**	**0.009**
MET	Hepatocyte growth factor receptor	1.060	0.290	1.074	0.412	**0.584**	**0.012**
ALCAM	CD166 antigen	1.030	0.540	0.973	0.574	**0.586**	**0.003**
EGFR	Epidermal growth factor receptor	1.053	0.236	1.026	0.579	**0.590**	**0.002**
LGALS3BP	Galectin-3-binding protein	0.983	0.816	0.826	0.120	**0.590**	**0.013**
TGFBI	Transforming growth factor-beta-induced protein ig-h3	1.016	0.720	0.836	**0.044**	**0.590**	**0.003**
PSMA2	Proteasome subunit alpha type-2	0.988	0.782	1.035	0.732	**0.591**	**0.017**
CTSH	Cathepsin H	1.000	0.992	1.071	0.425	**0.595**	**0.013**
EFNA2	Ephrin-A2	1.036	0.195	0.984	0.841	**0.600**	**0.010**
CAMK2B	Calcium/calmodulin-dependent protein kinase type II subunit beta	0.989	0.873	1.075	0.644	**0.600**	**0.017**
EPHA3	Ephrin type-A receptor 3	1.005	0.914	1.065	0.356	**0.600**	**0.016**
FYN	Tyrosine-protein kinase Fyn	0.970	0.492	1.030	0.643	**0.602**	**0.010**
TBP	TATA-box-binding protein	0.986	0.819	0.977	0.797	**0.603**	**0.013**
EPHB2	Ephrin type-B receptor 2	0.995	0.914	1.028	0.683	**0.603**	**0.016**
DKK4	Dickkopf-related protein 4	0.897	0.053	0.779	**0.032**	**0.604**	**0.016**
CSK	Tyrosine-protein kinase CSK	1.011	0.912	1.051	0.822	**0.609**	**0.008**
PLAUR	Urokinase plasminogen activator surface receptor	0.988	0.735	0.925	0.277	**0.615**	**0.008**
ADRBK1	beta-adrenergic receptor kinase 1	0.989	0.865	0.995	0.941	**0.616**	**0.021**
CTSB	Cathepsin B	0.978	0.622	1.123	0.120	**0.616**	**0.022**
HSPB1	Heat shock protein beta-1	1.010	0.819	1.007	0.951	**0.617**	**0.011**
CAMK2A	Calcium/calmodulin-dependent protein kinase type II subunit alpha	0.972	0.547	1.120	0.528	**0.617**	**0.020**
MCL1	Induced myeloid leukemia cell differentiation protein Mcl-1	0.984	0.679	0.941	0.401	**0.618**	**0.014**
ROBO2	Roundabout homolog 2	1.044	0.164	0.973	0.659	**0.626**	**0.011**
MATN2	Matrilin-2	0.992	0.825	1.037	0.649	**0.627**	**0.010**
CSRP3	Cysteine and glycine-rich protein 3	0.990	0.888	0.902	0.271	**0.628**	**0.011**
PDE11A	Dual 3′,5′-cyclic-AMP and -GMP phosphodiesterase 11A	1.022	0.716	0.997	0.920	**0.631**	**0.001**
TK1	Thymidine kinase, cytosolic	0.966	0.572	0.994	0.962	**0.633**	**0.028**
MAPK14	Mitogen-activated protein kinase 14	0.985	0.799	1.016	0.874	**0.635**	**0.004**
NRP1	Neuropilin-1	0.952	0.361	0.839	0.094	**0.636**	**0.018**
PIK3CA PIK3R1	PIK3CA/PIK3R1	0.982	0.828	0.972	0.802	**0.637**	**0.024**
PDPK1	3-phosphoinositide-dependent protein kinase 1	0.967	0.638	1.031	0.807	**0.639**	**0.029**
APOA1	Apolipoprotein A-I	1.000	0.991	1.106	0.228	**0.643**	**0.020**
SNX4	Sorting nexin-4	1.023	0.477	1.147	0.503	**0.645**	**0.047**
SLAMF7	SLAM family member 7	1.030	0.498	1.081	0.264	**0.646**	**0.016**
PPP3CA PPP3R1	Calcineurin	0.982	0.760	1.005	0.951	**0.651**	**0.009**
PRTN3	Myeloblastin	0.988	0.785	1.093	0.296	**0.651**	**0.019**
CCL15	C-C motif chemokine 15	1.000	0.996	1.121	0.205	**0.651**	**0.014**
GAS1	Growth arrest-specific protein 1	1.012	0.675	1.007	0.917	**0.652**	**0.032**
IL6ST	Interleukin-6 receptor subunit beta	1.021	0.468	0.907	0.241	**0.655**	**0.016**
LRPAP1	alpha-2-macroglobulin receptor-associated protein	1.047	0.084	0.991	0.794	**0.657**	**0.001**
LCN2	Neutrophil gelatinase-associated lipocalin	0.943	0.322	1.142	0.138	**0.657**	**0.016**
TNFRSF21	Tumor necrosis factor receptor superfamily member 21	1.023	0.435	0.968	0.720	**0.657**	**0.027**
CXCL6	C-X-C motif chemokine 6	1.029	0.448	0.975	0.788	**0.657**	**0.006**
MPL	Thrombopoietin Receptor	0.970	0.481	1.111	0.276	**0.659**	**0.017**
COL23A1	Collagen alpha-1(XXIII) chain	0.971	0.563	1.150	0.216	**0.659**	**0.042**
NLGN4X	Neuroligin-4, X-linked	0.978	0.587	1.022	0.822	**0.664**	**0.027**
SERPINE1	Plasminogen activator inhibitor 1	1.028	0.781	1.141	0.341	**0.664**	**0.029**
CCDC80	Coiled-coil domain-containing protein 80	1.063	0.145	0.969	0.689	**0.666**	**0.020**


*Values represent averages of all three biologic replicates and represent infected alterations compared to time-matched mock controls. Significance was determined by *T*-test and by *Z*-Score as detailed in Section “Materials and Methods” and values < 0.05 are **bolded**. Significant fold-changes > 1.50-fold at 12, 24, and 48 hpi are indicated in larger **bold** font in Fold-Change columns. Proteins are sorted by time point (early to late) with up-regulated at top and down-regulated below.*

To validate the SOMAScan results, we selected four cellular proteins predicted to have dysregulated expression by ZIKV infection and assessed their quantities at various times post infection. Fibronectin is involved in astrocytic cell growth, migration and actin polymerization, and STAT3 and SPARC are involved in astrocytic synaptic control. Commercial antibodies are available for each of these, and also for CST3 and were used to probe ZIKV-infected astrocytes (Figure 2B). Western blot confirmed that SPARC was significantly under-expressed at 24 hpi and that all proteins were under-expressed by 48 hpi (Figure 2B,C). There were apparent differences in the degree of SPARC expression as measured by both immunoblot and SOMAScan, but the Western blot and SOMAScan results trended in the same directions for all tested proteins. Minor differences in the extent of over/under-expression were observed between the two techniques (Figure 2C), but, overall, the immunoblot data validated the SOMAScan-measured protein dysregulation.

A global overview of these proteins with altered levels of expression, using IPA, indicates that cell death functions are highly activated at 48 hpi, while cell survival, cell outgrowth, cell migration, cell spreading and cell-to-cell contact functions are inhibited at 48 hpi (Figure 2D).

### ZIKV Differentially Disrupts Cellular Protein Levels and Pathways in Human Astrocytes and Monkey Kidney Cells

**FIGURE 3:**
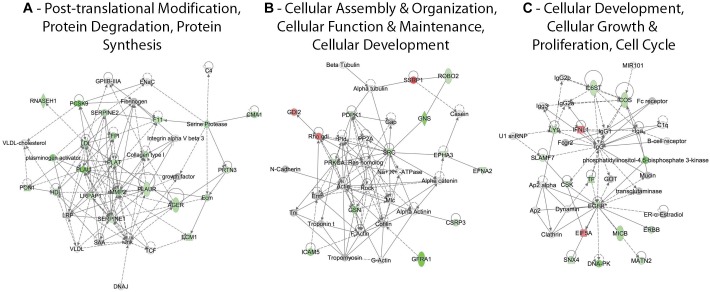
Significant differentially regulated U-251 protein networks. Proteins and their levels of regulation were imported into the Ingenuity Pathways Analysis (IPA^®^) tool and interacting pathways were constructed under default settings. Three of the top dysregulated U-251 cell networks that contain 12 or more “focus” molecules (molecules significantly up- or down-regulated) and that have network scores ≥ 20 at 48 hpi are identified. Red: significantly up-regulated proteins; pink: moderately up-regulated proteins; gray: proteins identified but not significantly regulated; light green: moderately down-regulated proteins; dark green: significantly down-regulated proteins; white: proteins known to be in network, but not covered within SOMAScan panel; dashed lines represent predicted or indirect interactions; solid lines represent direct known interactions. These networks, and top Vero cell differentially regulated networks, at 12, 24, and 48 hpi are shown in Supplementary Figures 1, 2.

Lists of the ZIKV-induced differentially expressed U-251 proteins were uploaded to Ingenuity Pathway Analysis (IPA) for network analysis. Multiple networks that contain ≥ 12 focus molecules and with scores ≥ 20 were identified. The IPA-generated scores for each network are based on the fit between the network and that of the set of focus genes identified in that network; this score is a measure of likelihood of finding those identified genes in that particular network by random chance (Long et al., 2004). A number of proteins involved in post-translational modification, protein degradation and protein synthesis were significantly under-expressed, with many more significantly under-expressed at later time points (Figure 3A). Molecules significantly over- or under-expressed in this network included transmembrane receptors [(AGER; receptor for advanced glycosylation end products), PLAUR], transporters (ECM1), various enzymes like peptidases (CMA1, F11, MMP2, PLAT, PLAU, PCSK9, and PRTN3) and phosphatases (PON1) and other molecules like RNASEH1, SERPINES 1 and 2 and TFPI (Figure 3A). Most molecules involved in cellular assembly, organization, maintenance and development are under-expressed except for GDI2, SSRP1 and Rho gdi (Figure 3B). Molecules significantly under-expressed in this network included kinases (PDPK1, EPHA3, EFNA2, PRKCA, and SRC), transmembrane receptors (GFRA1 and ROBO2) and other molecules (CSRP3, GDI2, GNS, GNS, ICAM5, and SSRP1) (Figure 3B). Cellular development, cellular growth and proliferation and cell cycle networks also had numerous over-/under-expressed proteins, with the majority being under-expressed (Figure 3C). Only IFNL1 and EIF5A were significantly over-expressed by 48 hpi. Molecules significantly under-expressed included kinases (CSK and EGFR), transmembrane receptors (ICOS, IL6ST and MICB), translation regulator (EIF5A), cytokine (IFNL1) and other molecules (LY9, MATN2, SLAMF7, SNX4, and TF) (Figure 3C).

We have previously reported SOMAScan^®^ analyses of ZIKV-infected monkey Vero cells (Glover et al., 2019). We therefore compared our differentially expressed protein lists using IPA. Three of the top U-251 networks (with ≥ 12 focus molecules and with scores ≥ 20) were: (1) Post-translational modification, protein degradation, protein synthesis; (2) Cellular assembly and organization, cellular function and maintenance, cellular development; and (3) Cellular development, cellular growth and proliferation, cell cycle. Numerous molecules within these networks were significantly under-expressed by ZIKV infection by 48 hpi. By contrast, while a few molecules (i.e., SSRP1, EIF5A, GSN, and SNX4) were similarly over- or under-expressed in both U-251 and Vero cells by 48 hpi, most of the differentially expressed U-251 proteins were not significantly affected in Vero cells (Supplementary Figure 1). Similarly, protein expression patterns impacted by ZIKV were different in the U-251 cells at 48 hpi compared to proteins present in the top two Vero cell networks (Supplementary Figure 2). For example, far more cell cycle, cell death and survival and cancer network proteins were over-expressed in Vero cell than in U-251 cells. In addition, while many proteins associated with the cancer, organismal injury and abnormalities and cellular movement network were similarly expressed in both cell types (i.e., IGFBP, CCL5 and TNFRSF21), several proteins (i.e., IGFBP3, ITGA1, and C4A/C4B) were dissimilarly impacted in the two cell types.

### ZIKV Impairs Numerous Astrocytic Canonical Pathways Including Axonal Guidance Signaling, FGF Signaling, STAT3 Signaling, AMPK and ERK/MAPK Signaling

**FIGURE 4:**
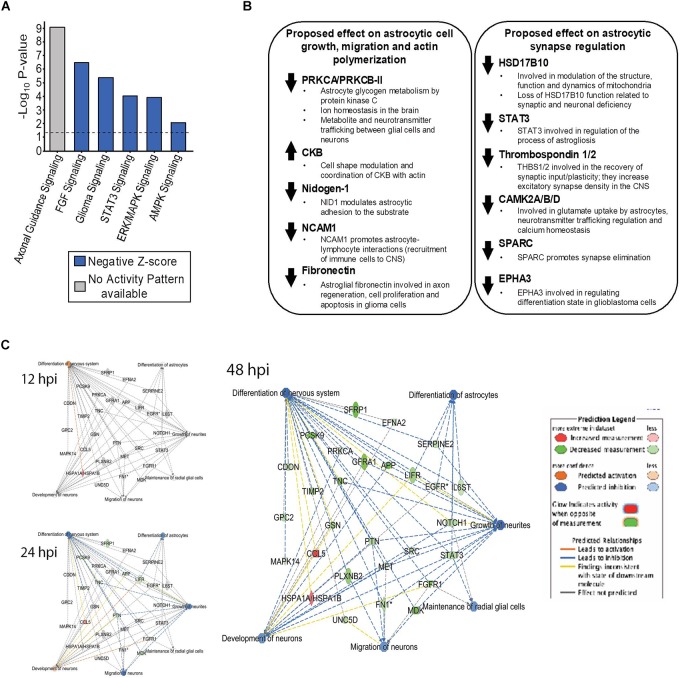
Dysregulated cellular molecules that potentially result in impairment of astrocytic functions crucial for astrocyte-mediated synaptic control, cell growth and migration. Differentially expressed proteins were imported into IPA and used to predict significant dysregulation of astrocyte/brain-specific functions. **(A)** Significantly dysregulated U-251 canonical pathways due to ZIKV infection by 48 hpi, along with their predicted activity pattern based on *Z*-score analyses. The *P*-values indicate the probability of association of molecules from the SOMA data set with each canonical pathway. Dotted line at –Log_10_ *P*-value of 1.3 indicates the cut-off value used. *Z*-scores describe the prediction in the overall activity of the canonical pathway based on the list of up/downregulated proteins identified from the SOMA list of proteins. **(B)** Up-/Down-regulation of individual molecules by ZIKV infection in U251 cells over time, along with the proposed astrocytic functions that are disrupted. **(C)** Predicted dysregulated functions known to result in numerous neurological disorders, described in the Introduction, that are associated with ZIKV infection.

Six canonical pathways; Axonal guidance signaling, FGF signaling, Glioma Signaling, STAT3 signaling, ERK/MAPK and AMPK signaling, which are highly important in astrocytic functions, were predicted to be dysregulated by ZIKV infection by 48 hpi (Figure 4A), with *Z*-scores exceeding ± 1.96. Most canonical pathways were predicted by IPA to be inhibited (blue bars), based upon the program considering the expression values of all identified proteins within each pathway. However, the most significantly dysregulated canonical pathway (axonal guidance signaling), could not be assigned by the program as overall activated or inhibited despite the large numbers of differentially expressed proteins (Table 3 and Supplementary Figure 3). Many of the molecules in these pathways control two major astrocyte functions; synaptic control and astrocytic cell growth and migration (actin polymerization) (Figure 4B). Protein Kinase C alpha/beta-II type (PRKCA/PRKCB-II), CKB, Nidogen-1, NCAM1 and fibronectin are involved in astrocytic cell growth, migration and actin polymerization (Figure 4B). HSD17B10, STAT3, Thrombospondin 1/2, CAMK2A/B/D, SPARC and EPHA3 are involved in astrocytic synaptic control (Figure 4B). SOMAScan indicated that CKB was over-expressed whereas PRKCA/PRKCB-II, Nidogen-1, NCAM1 and fibronectin were under-expressed by ZIKV by 48 hpi (Figure 4B). However, HSD17B10, STAT3, Thrombospondin 1/2, CAMK2A/B/D, SPARC and EPHA3 were under-expressed by ZIKV at 48 hpi (Figure 4B). Numerous differentially expressed astrocytic proteins also are predicted to inhibit differentiation of nervous system, differentiation of astrocytes, growth of neurites, maintain radial glial cells, neuron development and neuron migration by 48 hpi (Figure 4C).

**Table 3:** U-251 axonal guidance proteins significantly dysregulated by ZIKV by 48 hpi.

Entrez Gene Symbol	Protein	Fold Change	*P*-value	Location	Type
ADAM12	ADAM metallopeptidase domain 12	0.49	0.004	Plasma Membrane	Peptidase
EFNA2	Ephrin A2	0.60	0.010	Plasma Membrane	Kinase
EPHA3	EPH receptor A3	0.60	0.016	Plasma Membrane	Kinase
EPHB2	EPH receptor B2	0.60	0.016	Plasma Membrane	Kinase
FGFR1	Fibroblast growth factor receptor 1	0.20	0.000	Plasma Membrane	Kinase
FYN	FYN proto-oncogene, Src family tyrosine kinase	0.60	0.010	Plasma Membrane	Kinase
L1CAM	L1 cell adhesion molecule	0.34	0.001	Plasma Membrane	Other
MET	MET proto-oncogene, receptor tyrosine kinase	0.58	0.012	Plasma Membrane	Kinase
MMP2	Matrix metallopeptidase 2	0.46	0.003	Extracellular Space	Peptidase
NRP1	Neuropilin 1	0.64	0.018	Plasma Membrane	Transmembrane receptor
PLCG1	Phospholipase C gamma 1	1.81	0.004	Cytoplasm	Enzyme
PLXNB2	Plexin B2	0.34	0.001	Plasma Membrane	Transmembrane receptor
PRKACA	Protein kinase cAMP-activated catalytic subunit alpha	0.54	0.002	Cytoplasm	Kinase
PRKCA	Protein kinase C alpha	0.54	0.004	Cytoplasm	Kinase
PROK1	Prokineticin 1	0.39	0.003	Extracellular Space	Growth factor
ROBO2	Roundabout guidance receptor 2	0.63	0.011	Plasma Membrane	Transmembrane receptor
RTN4R	Reticulon 4 receptor	0.39	0.002	Plasma Membrane	Transmembrane receptor
UNC5C	UNC-5 netrin receptor C	0.39	0.002	Plasma Membrane	Transmembrane receptor
UNC5D	UNC-5 netrin receptor D	0.49	0.002	Plasma Membrane	Other


*Values represent averages of all three biologic replicates and represent infected alterations compared to time-matched mock controls. Significance was determined by *T*-test and by *Z*-Score as detailed in Section “Materials and Methods.” Only those proteins whose significant fold-change was ≥ 1.50-fold in either direction (i.e., ≥1.50 or ≤0.666) are shown.*

### ZIKV Infection Leads to the Dysregulation of Numerous Upstream Cellular Regulator Molecules in U251 Cells by 48 hpi

**FIGURE 5:**
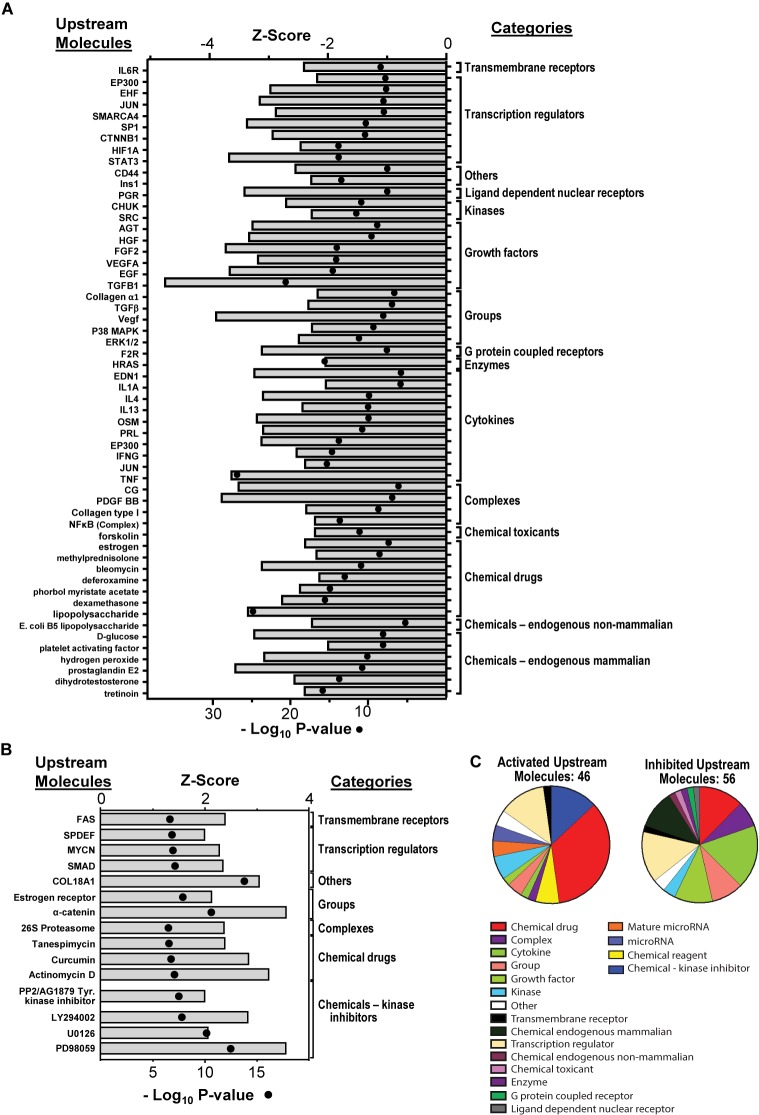
Dysregulation of upstream molecules that control downstream pathways, regulators, and gene expression. **(A)** Predicted inhibition of upstream regulators dysregulated by ZIKV at 48 hpi; all molecules had a *p*-value < 10^-7^ and are predicted to be inhibited as shown. **(B)** Predicted activation of upstream regulators dysregulated by ZIKV at 48 hpi. The molecules that had a *p*-value of < 10^-6^ and are predicted to be activated are shown. *P*-values are shown as dots while *Z*-scores are shown as bars. **(C)** Overall distribution of different types of upstream molecules dysregulated by ZIKV at 48 hpi. All these molecules are predicted to be significant based on IPA analysis.

Based on the over- and under-expression of a number of downstream molecules, 56 upstream molecules were identified and their activity was predicted to be significantly inhibited, and 46 upstream molecules were identified and predicted to be significantly activated by ZIKV by 48 hpi (Figure 5). These upstream molecules are predicted by the IPA program and therefore not necessarily probed by SOMAScan. Based on the over-/under-expression of each of their downstream molecules, that are probed by SOMAScan, IPA generates a prediction of the activation status of these upstream molecules. Therefore, even though the expression of some of these upstream molecules was not directly examined by the SOMAScan, IPA was able to predict their activation status (with high significance; i.e., *p*-values < 10^-7^ for inhibited and <10^-6^ for activated). These molecules belong to numerous categories including transmembrane receptors, transcription regulators, ligand-dependent nuclear receptors, growth factors, cytokines and G protein coupled receptors. A subset of these molecules, and the implications of their dysregulation on astrocytic regulation of nervous system organization and differentiation (Landstrom et al., 2000; Mazars et al., 2001) will be further discussed below in the Section “Discussion.”

## Discussion

We employed a novel proteomic approach to assess ZIKV-induced alterations in levels of U-251 astrocyte protein expression during a time course after infection. We showed that ZIKV can successfully infect and replicate in U-251 cells and that under our infection conditions (MOI = 3), >90% of the cells were infected by 48 hpi (Figure 1E), allowing meaningful proteomic analyses. The vast majority of proteins were significantly under-expressed, especially by late time points, and many of these differentially expressed proteins belong to specific astrocyte cell functions, upstream complexes/group of molecules/transcriptional regulators, and several individual key molecules (Figure 2). A few of these cellular functions and molecules, such as inhibition of differentiation, cell growth dysregulation and modulation of stress responses, have been identified in other ZIKV-infected cell types, including Vero cells, C6/36 cells and human neural stem cells (Devhare et al., 2017; Hou et al., 2017; Glover et al., 2019).

One of the dysregulated cellular systems was the Post-translational modification, protein degradation and synthesis network (Figure 3A). Molecules significantly over- or under-expressed in this network included transmembrane receptors [AGER (Receptor for advanced glycation end products), PLAUR], transporters (ECM1), various enzymes like peptidases (CMA1, F11, MMP2, PLAT, PLAU, PCSK9, and PRTN3) and phosphatases (PON1) and other molecules like RNASEH1, SERPINES 1 and 2 and TFPI (Figure 3A). They localize in the nucleus, plasma membrane and extracellular space. Their differential expression is known to impair a number of astrocytic functions like apoptosis and astrocytic reactivity. For instance, down-regulation in the expression of AGER is associated with modulation of cell apoptosis, localization of NF-κB, phosphorylation of ERK1/2, and astrocyte reactivity leading toward neurodegeneration (Wang et al., 2002; Ohtaki et al., 2007; Choi et al., 2014; Kim et al., 2015; Gu et al., 2016). Differential expression of other molecules like MMP-2 (Matrix Matalloproteinase-2) and SERPINES 1/2 (Serpin family E member 1 and 2) results in the proteolytic cleavage of pericellular proteins and breakdown of extra-cellular matrix, stalls cell migration and neurite remodeling in the brain, which in turn can impact brain development (Ogier et al., 2006; Hagemann et al., 2012; Kamat et al., 2014; Wu et al., 2018; Yang et al., 2018).

Another dysregulated cellular system was the Cellular assembly and organization, cellular function and maintenance and cellular development network. Molecules significantly affected in this network included kinases (PDPK1, EPHA3, EFNA2, PRKCA, and SRC), transmembrane receptors (GFRA1 and ROBO2) and other molecules (CSRP3, GDI2, GNS, GNS, ICAM5, and SSRP1) (Figure 3B). These are localized in the nucleus, cytoplasm, plasma membrane and extra cellular space. They control various astrocytic cellular functions. For example, PDPK1 (3-phosphoinositide dependent protein kinase 1) dysregulated expression is known to damage glucose metabolism, cell migration, cell survival and neural plasticity (Scheid et al., 2005; Park et al., 2013). Under-expression of PRKCA (Protein kinase C alpha) is linked with modulation of apoptotic signaling and cell proliferation while Src (SRC proto-oncogene, non-receptor tyrosine kinase), which has a role in actin dynamics in U-251 cells, is associated with the disruption of cell migration (Angers-Loustau et al., 2004; Marquina-Sanchez et al., 2017). In addition, the under-expression of GFRA1 (GDNF family receptor alpha 1) by ZIKV can disrupt its binding with GDNF (glial cell-line derived neurotrophic factor), dysregulating the process of cell differentiation and proliferation indirectly (Takashima et al., 2015; Ayanlaja et al., 2018).

A third major dysregulated cellular system was the Cell development, cell growth, proliferation and cell cycle network. The molecules significantly affected in this network included kinases (CSK and EGFR), transmembrane receptors (ICOS, IL6ST, and MICB), translation regulator (EIF5A), cytokine (IFNL1) and other molecules (LY9, MATN2, SLAMF7, SNX4, and TF) (Figure 3C). These are localized in the cytoplasm, plasma membrane and extracellular space. Their abnormal expression leads to disruption of numerous astrocytic cellular functions. For example, under-expression of EGFR (Epidermal growth factor receptor) is known to result in dysregulation of the formation of reactive astrocytes and negatively impact EGFR-mediated cell migration (Liu and Neufeld, 2007). Since EGFR controls astrocytic survival and differential growth in cortical and midbrain astrocytes, its under-expression by ZIKV may result in differential apoptotic responses in various regions of the brain (Wagner et al., 2006; Liu and Neufeld, 2007) Disruption in EGFR is also linked with the disorganization of axons, fewer cell-to-cell contacts between neurons and astrocytes and apoptosis induction, resulting in the state of neurodegeneration (Wagner et al., 2006; Liu and Neufeld, 2007). In addition, EGFR serves as a coreceptor for facilitating entry of influenza A virus (IAV) and other flaviviruses, such as Hepatitis C virus (HCV) (Lupberger et al., 2013; Zheng et al., 2014), and EGFR also regulates interferons via STAT1 (Lupberger et al., 2013; Zheng et al., 2014). ZIKV also caused under-expression of STAT3 in our U-251 cells. Collectively, these imply ZIKV may modulate the EGFR signaling pathway and host proinflammatory response to promote its successful replication. The eukaryotic translation factor eIF5A is another up-regulated molecule, which is involved in translation elongation and termination beyond the ribosome pause state due to polyproline motifs (Pelechano and Alepuz, 2017; Schuller et al., 2017). This seems plausible since ZIKV needs to hijack the cellular protein translation machinery for production of its progeny proteins. Moreover, other eukaryotic translation factors that were significantly up-regulated by ZIKV infection, although only ≈30%, include eIF4H and eIF5A-1. Another aspect of eIF5A that should be considered is the hypusination process that it undergoes as part of its unique post-translational modification; this process is critical for its function and is important for mRNA stability (Olsen and Connor, 2017). Furthermore, since other flaviviruses like dengue virus are sensitive to utilizing polyamines, needed for the hypusination process (Olsen and Connor, 2017; Smirnova et al., 2018), ZIKV-induced over-expression of eIF5A in U-251 cells seems plausible. Comparative analyses with the Vero cells at 48 hpi also showed a few of these same differentially expressed proteins; namely eIF5A, C4, SSRP1, EPHA3 and PDPK1 (Supplementary Figure 1). In addition to overlaying ZIKV-infected Vero cell data on our top ZIKV-infected U-251 networks, we also overlaid U-251 data onto the top identified dysregulated Vero cell networks (Supplementary Figure 2). Once again, a number of molecules, including HSPA1A/B, PARK7, HMGN1, IGF1R, IGFBP2/5, L1CAM and CCL5 were similarly over- or under-expressed in both systems (Supplementary Figure 2). All of these impairments of astrocytic cellular differentiation, migration and effect on neurite growth via dysregulated astrocytic functions were also significantly predicted by the IPA program (Figure 4). In addition, a few of the highly crucial canonical pathways needed in order for astrocytes to function normally were also predicted to be inhibited by IPA (Figure 4).

In addition to astrocyte cell migration, reactivity and differentiation, IPA also predicted ZIKV impacting their interaction with neural cells (Reemst et al., 2016). The formation of contact between astrocytes and neural synapses is necessary for the establishment of the “tripartite synapse,” brain homoeostasis, synaptic transmission regulation and supplying substrates to the neurons (Reemst et al., 2016; Becerra-Calixto and Cardona-Gomez, 2017). Astrocytic cell movement and outgrowth are important during the developmental stage when astrocytes need to migrate to various sites in the CNS which later become their final destination (Chaboub and Deneen, 2013). Both of these functions were predicted to be downregulated by ZIKV (Figure 2D,4C). Several important individual molecules highly involved in these processes and that are highly under-expressed include protein kinase C (PRKCA/PRKCB-II), which aids adhesion of astrocytes to the local neurons; thrombospondins 1 and 2 (TSP1, TSP2), which drive synapse assembly, aid recovery of synaptic plasticity and increase excitatory synapse density in the CNS; and SPARC, which is a negative regulator of maturation of presynaptic termini and promotes synapse elimination (Figure 5B) (Hama et al., 2004; Allen, 2013; Terni et al., 2017). PRKACA and PRKCA also are involved in axonal guidance (Figure 4A, Table 3, and Supplementary Figure 3). Numerous other proteins like MMP2, EPHA3, EFNA2, and FGFR1 which are involved in cellular assembly, organization, astrocytic reactivity, and adhesion of astrocytes to local neurons also regulate the axonal guidance pathways (Table 3). Despite the fact that IPA was unable to generate a prediction for this canonical pathway as either activated or inhibited, the list of 19 highly under-expressed proteins points toward dysregulation in the overall pathway (Figure 4A). Further detailed prediction patterns were also generated based on the expression profiles of proteins involved in the axonal guidance pathway and they point toward both inhibition and activation of axon dynamics, repulsion, outgrowth, and growth cone spreading (Supplementary Figure 3). Overall, this ZIKV-induced dysregulation at both the expression level and the predicted activity level highlights another way ZIKV may disrupt astrocytic function, leading toward overall brain conditions like microcephaly. FN1 (fibronectin) and laminin are another set of ZIKV-induced differentially expressed proteins in astrocytes that are important in the elongation of axons and pathfinding via contact-mediated attraction of the growth cone (Figure 5B) (Reemst et al., 2016). Moreover, CKB (creatine kinase B-type), which was highly over-expressed by ZIKV infection, is crucial in maintaining ion homeostasis in the brain, and glutamate uptake in neurotransmitter trafficking between astrocytes and oligodendrocytes and between axons and the contact points with the nodes of Ranvier (Steen et al., 2010). This ZIKV-induced differential expression in CKB levels could also disrupt the astrocyte function of maintaining glutamate toxicity for local neurons (Steen et al., 2010). Since CKB also co-localizes with actin and helps in astrocytic cell spreading, its under-expression by ZIKV could explain impairment in cell spreading and migration (Kuiper et al., 2009). Glutamate uptake by astrocytes is also controlled by CAMKII and our SOMAScan data showed its under-expression by ZIKV infection at 48 hpi, in part explaining the dysregulation in neurotransmitter trafficking via modulation of CAMKII (Figure 5B) (Chawla et al., 2014). These CAMKII fluctuations are also known to induce neuronal death by disrupting calcium homoeostasis (Ashpole et al., 2012, 2013). There were numerous other differentially expressed targets shown in Figure 4 along with the proposed astrocytic function that could be impaired by ZIKV-induced dysregulation (Yang et al., 2009, 2014; Tyzack et al., 2014). Thus, these multiple impaired functions help explain effects which may lead toward neuronal damage, neuronal and astrocytic degeneration and overall cell death resulting in conditions like microcephaly or other disorders (Wen et al., 2017).

IPA analyses also predicted activation/inhibition of various upstream regulators that could stall a variety of cellular canonical pathways (Figure 5). They belong to numerous broad classes like transmembrane receptors, transcription regulators, growth factors, cytokines, kinases and others (Figure 5C). These upstream molecules are generated by the IPA program and are not necessarily examined by SOMAScan. Based on the over/under-expressed of each of their downstream molecules, that are probed by SOMAScan, IPA generates a prediction on the activation status of these upstream molecules. Therefore, even though the expression of some of these upstream molecules was not directly examined by the SOMAScan, IPA was able to predict their activation status (with high significance; i.e., *p*-values < 10^-7^ for inhibited and <10^-6^ for activated). Signal transducer and activator of transcription-3 (STAT3) is a transcriptional regulator that was predicted to be inhibited (Figure 5A) in addition to being under-expressed by ZIKV (Figure 4C). Since it is involved in various key aspects of astrogliosis (promoting formation of reactive astrocytes) and astrocyte migration, it helps explain ZIKV-induced dysregulation leading to the abnormal production of reactive astrocytes, most of which are related to glial scar formation (Okada et al., 2006; Ceyzeriat et al., 2016; Renault-Mihara et al., 2017). In addition, STAT3 also has a role in type 1 interferon signaling pathway; its under-expression can result in modulation of the antiviral activity/response due to IFN type 1 (Ho and Ivashkiv, 2006; Hong and Song, 2014; Suarez et al., 2018). Interferon type I (especially IFNα) activates the STAT3 gene which in turn attenuates type I IFN inflammatory responses (Ho and Ivashkiv, 2006). This STAT3-induced negative regulation of type I IFN response has been shown in mice models where a STAT3 KO resulted in an increased IFN type I production upon viral infection (Wang et al., 2011). This enhanced immune response stimulates expression of a number of ISGs, including ISG15 (Wang et al., 2011), which we found was over-expressed 2.75-fold by 48 hpi (Table 2). Deficiency in IFN-α/β receptor (IFNAR) is also known to make mice highly susceptible to ZIKV infection (Yockey et al., 2018). Since interferon type I activates STAT3, the decrease in STAT3 expression in our data set can in part be explained with the under-expression of IFNA2 (Table 2). To better understand the functions of STAT3, analyses of STAT3 activity and its modulation in response to ZIKV-induced IFNA2 under-expression seems like a next plausible approach. Given recent research showing induction of alpha/beta interferon (IFN-α/β) in response to coronavirus infection in the CNS, which is followed by up-regulation of various interferon stimulating genes (ISGs), the impact of ZIKV infection on the expression of ISGs can in part be explained by modulation in the IFN type 1 responses (Hwang and Bergmann, 2018). The observed increase in ISG15 due to ZIKV infection at 48 hpi in U251 cells is consistent with the findings on ISG15 expression modulation due to other flaviviruses like Dengue virus (DENV) and West Nile Virus (WNV) (Dai et al., 2011). Since ISG15 has such an important immunomodulatory role, its over-expression and antiviral roles have also been linked with other viruses like Hepatitis E virus (HEV), influenza A/B, herpes simplex virus type 1, and HIV (Dai et al., 2011; Sooryanarain et al., 2017). Furthermore, other interferons, such as interferon lambda 1 (IFNL1) were also over-expressed due to ZIKV infection by 48 hpi (Table 2). IFN-λ is already known to inhibit ZIKV infection in the female reproductive tract (Caine et al., 2019). Additional studies focusing on each of these types of interferon I, II, and III, and their interplay with each other and with STAT3 in response to ZIKV infection, could help explain viral-induced host responses.

The 26S proteasome complex was another upstream regulator predicted to be activated by ZIKV infection (Figure 5B). The 26S proteasome complex is necessary for degradation of target proteins tagged by multiple ubiquitin markers, utilizing the ubiquitin conjugation cascade and the proteolytic properties of the proteasome core (Myung et al., 2001; Baert et al., 2007). Thus, it is likely that the proteasome, which would normally use this protein degradation process to remove misfolded proteins from the cellular system, is turned on by ZIKV to aid its replication. Further study to fully understand the relationship between the activated 26S signaling pathway and the production of viral proteins is warranted. The PI3K complex also was predicted to be inhibited by ZIKV. The predicted inhibition of PI3K complex results in dysregulation of various aspects of cell function like cell survival, cell proliferation, cellular trafficking, protein translation and RNA processing, since the PI3K complex lies upstream of most of these cellular functions’ respective molecular pathways (Wang L. L et al., 2017; Ahmad and Siddiqui, 2018). One of its downstream molecules is AKT, RAC-alpha/beta/gamma serine/threonine-protein kinase, and its downregulation by ZIKV is known to modulate glucose metabolism, cell differentiation, regulation of gene transduction and apoptosis (Wang L. L et al., 2017; Ahmad and Siddiqui, 2018). The ERK1/2 (Extracellular signal-regulated kinase 1/2) and MAP2K1 (Dual specificity mitogen-activated protein kinase 1) molecules were another set of molecules predicted to be inhibited by ZIKV (Figure 4, 5). These proteins also are modulated by other flaviviruses (Cheng et al., 2018). For example, HCV modulates the Ras/Raf/MEK pathways to attenuate the JAK-STAT pathway and induce viral replication, YFV and DENV-2 both induce MAPK and ERK1/2 pathways respectively to promote their replication (Cheng et al., 2018). In addition, MEK/ERK signaling is involved in modulating astrocytic differentiation and an inhibition of these molecules is associated with the impairment of cell differentiation and modulation of astrocyte reactivity (Mandell and VandenBerg, 1999; Li et al., 2012). This predicted inhibition also is linked with disruption in neuronal support and synaptic functioning, two crucial functions of normally differentiated astrocytic cells (Mandell and VandenBerg, 1999; Li et al., 2012). Some of these ZIKV-mediated alterations on the infected U-251 cellular proteome also were observed in our recent study of ZIKV-infected Vero cells (Glover et al., 2019). For example, ZIKV down-regulated STAT3 and up-regulated eIF5A expression in both cell systems (Glover et al., 2019). In Vero cells, ZIKV induced activation of the Tec Kinase pathway, linking it to Ca+2 influx, cell proliferation, differentiation, migration and apoptosis, showing that even though the underlying effect ZIKV has on the molecular profile of Vero cells varies from that of U-251 cells, the effect at the functional level has some similarities (Glover et al., 2019). Recognizing these similarities and differences in the protein expression levels in response to ZIKV infection in both U251 and Vero cells are very important and relevant. Since the Vero cell line still is the most commonly used cell line to test a number of antiviral drugs, it is crucial to understand how well the proteomic profile of ZIKV infection in U251 cells, which is a more physiologically relevant cell type, compares to that in the Vero cell type (Perera et al., 2008; Farias et al., 2013; Zamora et al., 2016; Wang S. B et al., 2017; Haviernik et al., 2018). This way, eventual translation of the large number of antiviral drugs and vaccines that have already been tested in Vero cells against a number of other flaviviruses like Dengue Virus Type 2 and West Nile Virus, into the ZIKV system can be made more efficiently (Perera et al., 2008; Farias et al., 2013; Zamora et al., 2016).

## Conclusion

In conclusion, ZIKV successfully infects and replicates in astrocytic U-251 cells. A detailed proteomic analysis of the ZIKV-induced dysregulated cellular proteins revealed numerous dysregulated networks and cellular functions, especially those pertaining to astrocytic functions in the brain, and upstream regulator molecules that provide more explanation of how ZIKV can cause neurodegeneration and impairment of neural circuit and network development resulting in conditions like microcephaly. Extension of similar studies to other cell types infectable by ZIKV are warranted to develop a fuller comprehension of ZIKV-induced proteomic alterations that could be targeted for therapeutic intervention.

## Data Availability

All datasets generated for this study are included in the manuscript and/or the Supplementary Files.

## Author Contributions

AS performed most of the experiments, interpreted the results, and wrote the manuscript first draft. KG assisted in experimental set-up, in results interpretation, and edited the manuscript. KC provided overall guidance, interpreted the results, and edited the manuscript.

## Conflict of Interest Statement

The authors declare that the research was conducted in the absence of any commercial or financial relationships that could be construed as a potential conflict of interest.
