# Supplementary material for: Zika Virus Infection Disrupts Astrocytic Proteins Involved in Synapse Control and Axon Guidance
Source: Front Microbiol. 2019 Mar 26;10:596. doi: 10.3389/fmicb.2019.00596 (PMC6448030; doi:10.3389/fmicb.2019.00596)
Supplement: Supplementary file 1 [file Image_1.pdf]

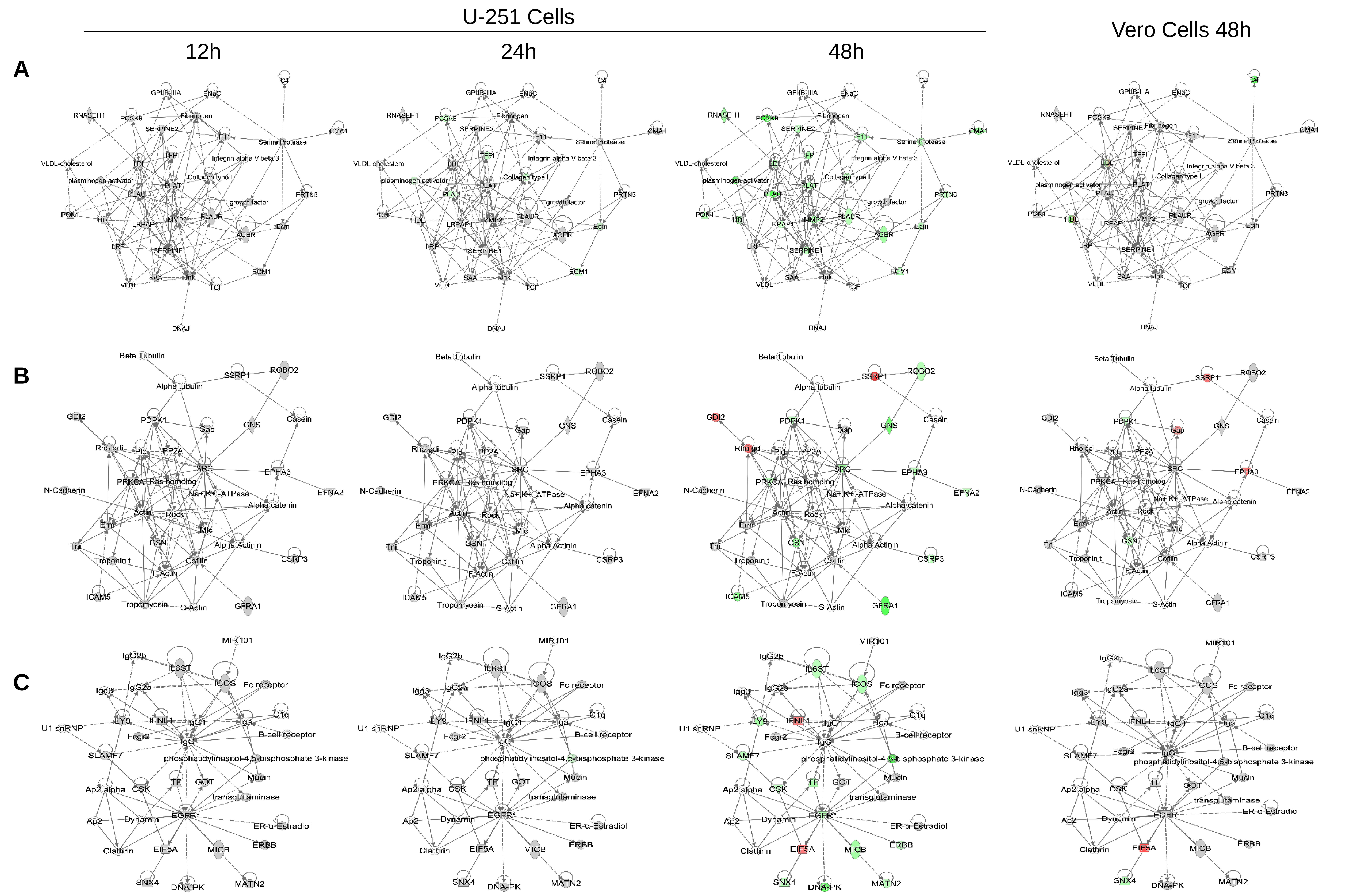

Supplementary Figure 1. Significantly dysregulated U-251 protein networks. Proteins and their levels of regulation were imported into the Ingenuity Pathways Analysis (IPA®) tool and interacting pathways were constructed. Three of the top networks that contain 12 or more "focus" molecules (molecules significantly up- or down-regulated) and that have network scores ≥ 20 at 48 hpi are identified. **A,** Post-translational modification, protein degradation and synthesis; **B,** Cellular Assembly and Organization, Cellular Function and Maintenance, Cellular Development; **C,** Cellular development, cellular growth and proliferation and cell cycle. ZIKV-induced cellular protein dysregulation at 12, 24 and 48 hpi in the U-251 cells are indicated at top. Dashed lines represent predicted or indirect interactions; solid lines represent direct known interactions; red: significantly up-regulated proteins; pink: moderately up-regulated proteins; grey: proteins identified but not significantly regulated; light green: moderately down-regulated proteins; dark green: significantly down-regulated proteins; white: proteins known to be in network, but not covered within SOMAScan panel. Vero cell proteins dysregulated within these networks at 48 hpi (as determined by (Glover et al., 2018)) are shown at far right for comparative purposes.
