# Supplementary material for: Zika Virus Infection Disrupts Astrocytic Proteins Involved in Synapse Control and Axon Guidance
Source: Front Microbiol. 2019 Mar 26;10:596. doi: 10.3389/fmicb.2019.00596 (PMC6448030; doi:10.3389/fmicb.2019.00596)
Supplement: Supplementary file 2 [file Image_2.pdf]

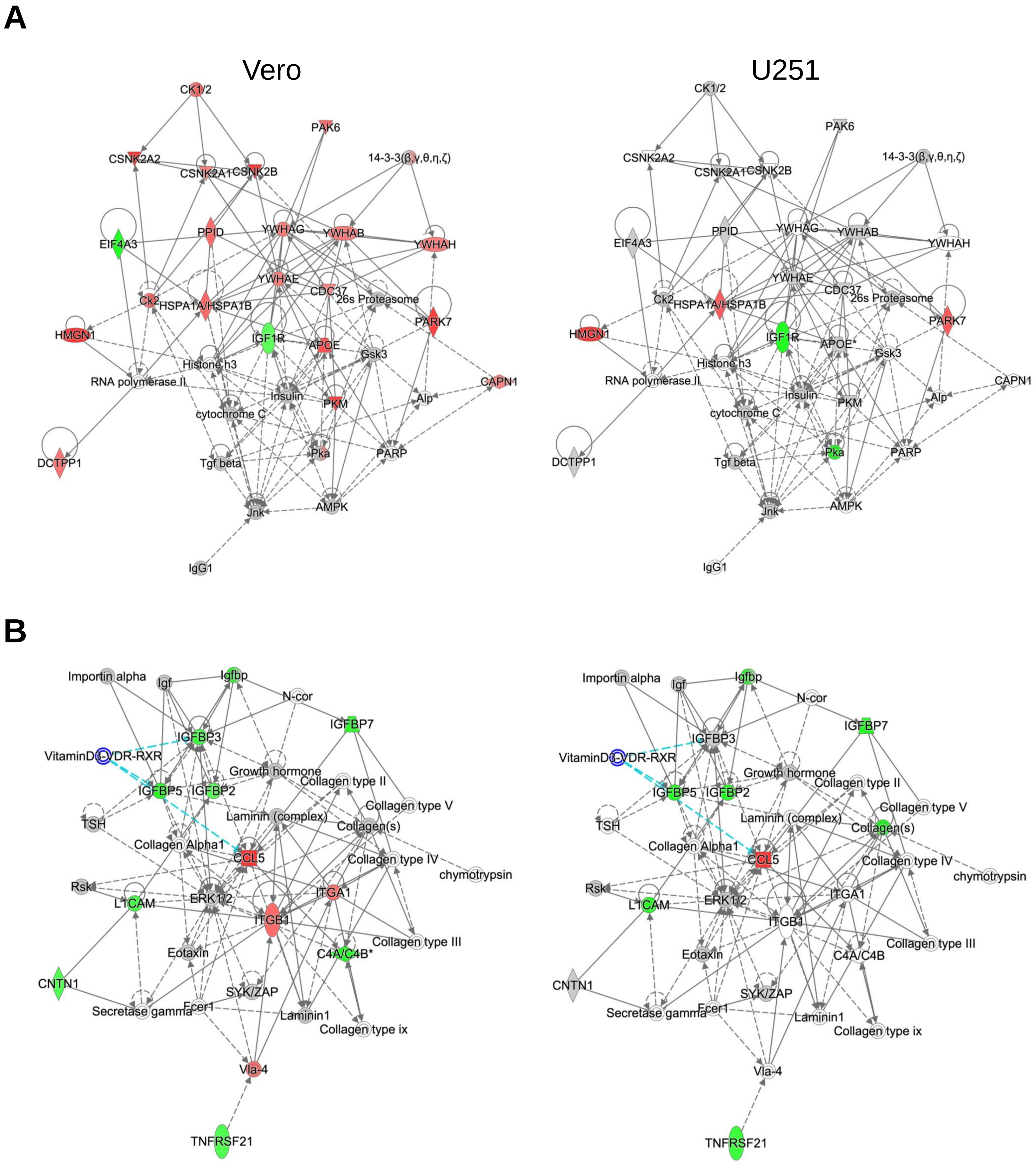

Supplementary Figure 2. Significantly dysregulated Vero cell protein networks. The top two IPA-generated Vero cell networks dysregulated by ZIKV at 48 hpi (from Glover et al., 2018) were overlaid with the proteomic data from the ZIKV-infected U-251 cells at 48 hpi to highlight the similarities and differences in the two cell lines. **A,** Cell cycle, cell death and survival, and cancer; **B,** Cancer, organismal injury and abnormalities, and cellular movement. Red: significantly up-regulated proteins; pink: moderately up-regulated proteins; grey: proteins identified but not significantly regulated; light green: moderately down-regulated proteins; dark green: significantly down-regulated proteins; white: proteins known to be in network, but not covered by SOMAScan panel; dashed lines: predicted or indirect interactions; solid lines: direct known interactions.
