# Supplementary material for: Zika Virus Infection Disrupts Astrocytic Proteins Involved in Synapse Control and Axon Guidance
Source: Front Microbiol. 2019 Mar 26;10:596. doi: 10.3389/fmicb.2019.00596 (PMC6448030; doi:10.3389/fmicb.2019.00596)
Supplement: Supplementary file 3 [file Image_3.pdf]

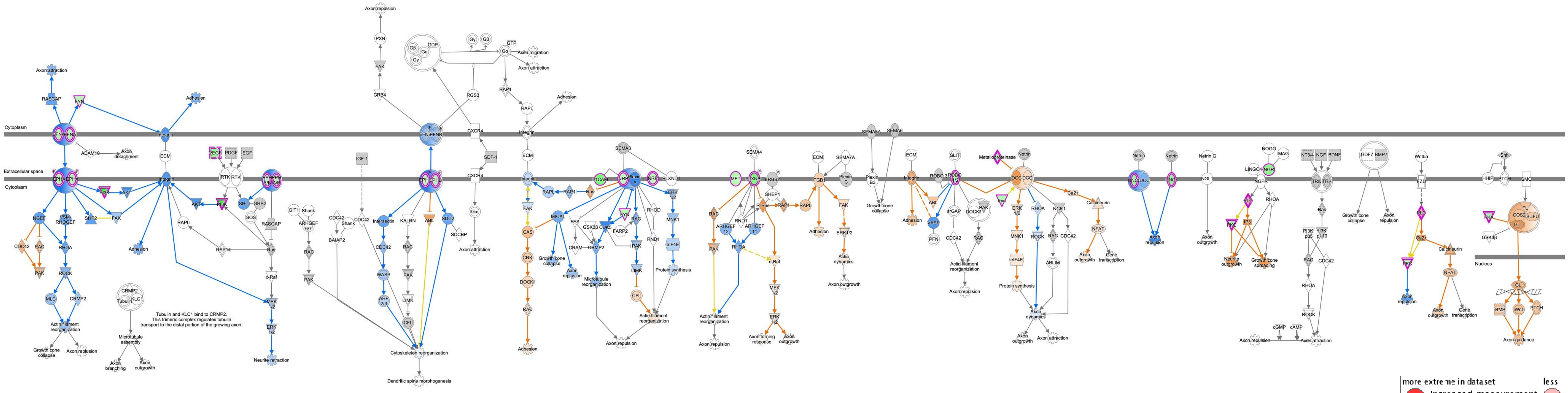

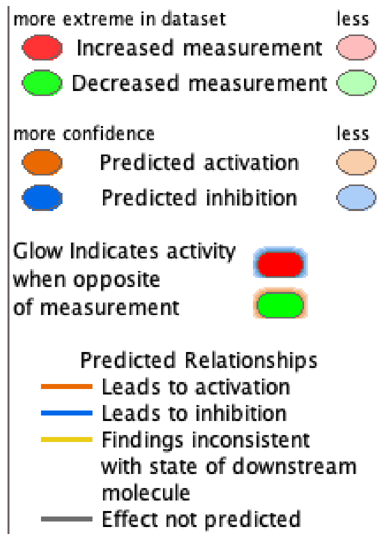

Supplementary Figure 3. The Axonal Guidance Canonical Pathway. Significantly differentially expressed U-251 proteins are indicated. Color coding is indicated in legend at right; white: proteins known to be in pathway, but not covered by SOMAScan panel.
